# Spherical pneumonia caused by *Ralstonia mannitolilytica*: a case report and literature review

**DOI:** 10.1186/s12890-023-02316-8

**Published:** 2023-01-16

**Authors:** Jianli Ma, Chuantao Zhang, Kaijie Dang, Yichao Liao, Xue Feng, Pengcheng Zhou

**Affiliations:** 1 Clinical Medical School, Chengdu University of Traditional Chinese Medicine, No 39 Shi-Er-Qiao Road, Jin Niu District, Chengdu, 610072 Sichuan Province People’s Republic of China; 2 Department of Respiratory Medicine, Hospital of Chengdu University of Traditional Chinese Medicine, Chengdu, Sichuan Province People’s Republic of China

**Keywords:** Spherical pneumonia, *Ralstonia mannitolilytica*, Metagenomic next-generation sequencing, Case report

## Abstract

**Background:**

Spherical pneumonia is an extremely rare condition that is difficult to diagnose. It is a specific type of lung infection that often manifests as a round or round-like mass on chest imaging. Spherical pneumonia is easily misdiagnosed as a pulmonary tumor; therefore, awareness of this disease must be strengthened.

**Case presentation:**

The patient was a 29-year-old female who had persistent cough and sputum for approximately 1 month and fever for 5 days. Chest computed tomography (CT) at our hospital revealed a mass in the lower lobe of the right lung near the hilar region, with obstructive pulmonary atelectasis and obstructive pneumonia. Although lung cancer was suspected, *Ralstonia mannitolilytica* was detected by metagenomic next-generation sequencing (mNGS) of bronchoalveolar lavage fluid, and no cancer cells or *Mycobacterium tuberculosis* were detected. Finally, the patient was diagnosed with spherical pneumonia caused by *R. mannitolilytica*. Anti-infective treatment, symptomatic treatment, and administration of a traditional Chinese medicine decoction were performed based on the syndrome differentiation. After 10 days of treatment, chest CT revealed few lesions in the lower lobe of the right lung, which were significantly reduced compared with those in the past.

**Conclusions:**

Spherical pneumonia caused by *R. mannitolilytica* has not yet been reported and differential diagnosis is key in clinical diagnosis. When spherical pneumonia is difficult to diagnose, mNGS may be a better alternative.

## Background

Spherical pneumonia is a rare and specific type of lung inflammation that most commonly presents as a round or round-like mass shadow on imaging [1]. It is difficult to diagnose clinically and is often misdiagnosed as lung cance [2]. The clinical presentation of spherical pneumonia is atypical; patients may have respiratory symptoms such as fever, cough, sputum, hemoptysis, and chest pain, or they may have no symptoms and are often discovered by physical examination [3]. Although the pathogenic composition of spherical pneumonia is currently considered similar to that of community-acquired pneumonia, atypical or rare pathogens continue to be reported, as detection techniques continue to advance [4]. The success of spherical pneumonia treatment is closely related to the timely identification of the causative agent and targeted anti-infective therapy, and identification of the pathogenic agent is a prerequisite for the clinical treatment. Herein, we report a case of spherical pneumonia caused by *Ralstonia mannitolilytica* for the first time.

## Case presentation

**Fig. 1:**
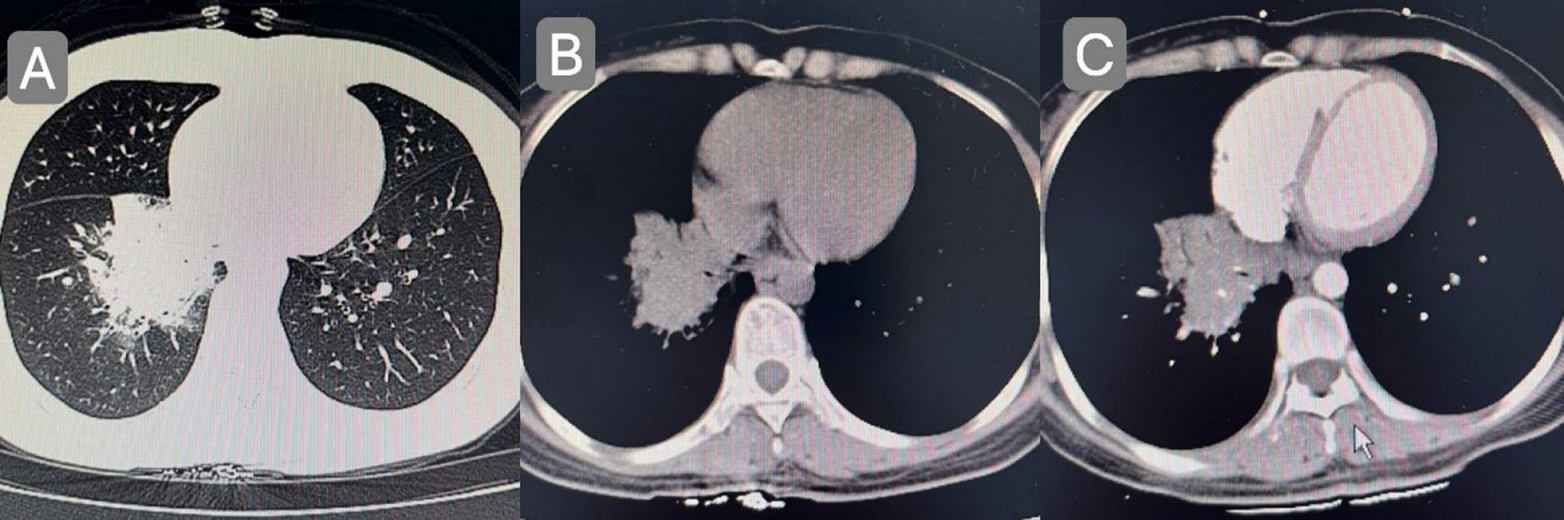
**A**, **B** Computerized tomography scan showing mass shadow in right lower lobe near hilar area with obstructive atelectasis and obstructive pneumonia. **C** Computerized tomography enhanced scan showing mass shadow near the hilar area of the right lower lobe, suggesting the possibility of central lung cancer, accompanied by obstructive pneumonia changes

**Fig. 2:**
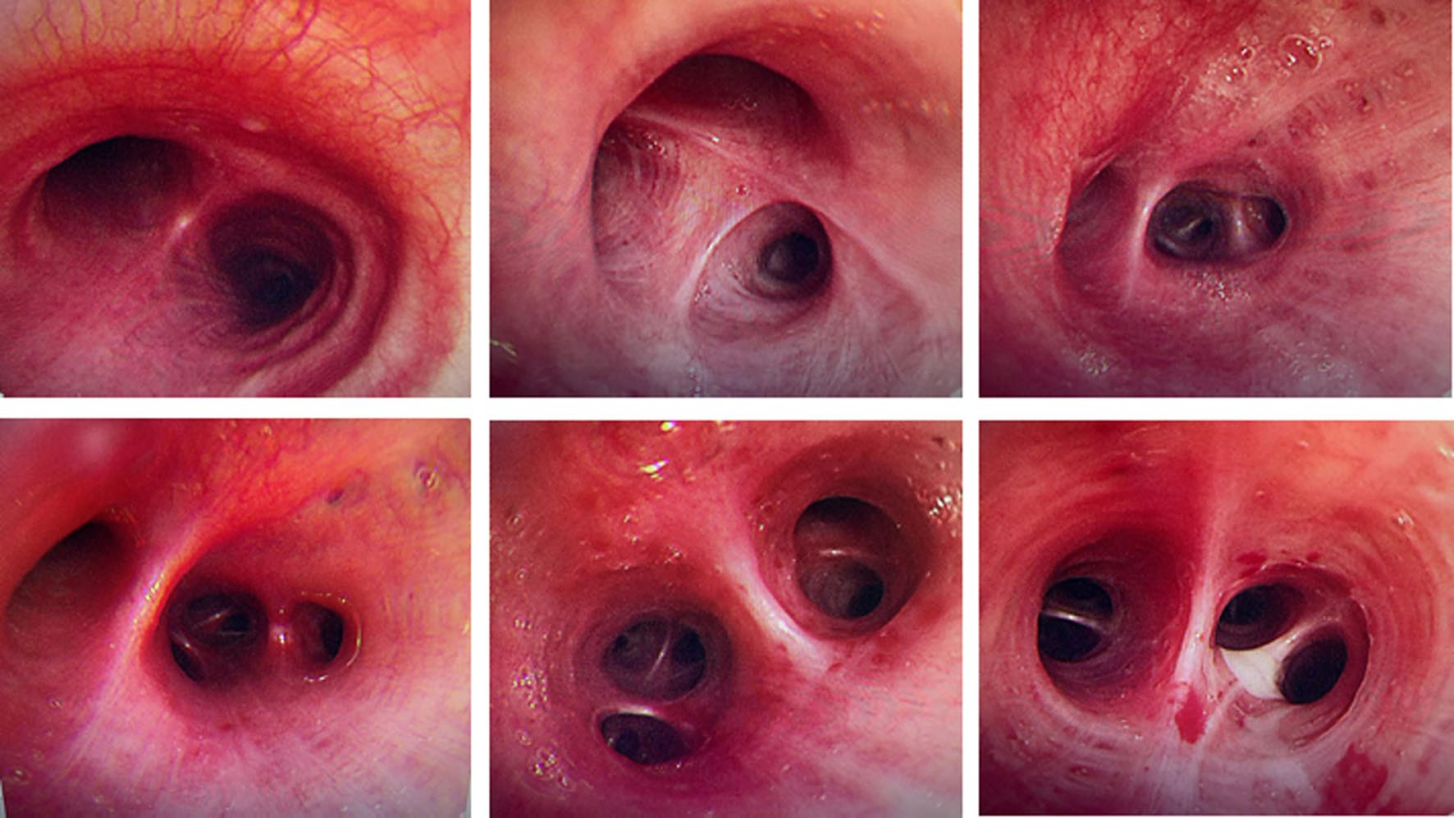
Bronchoscopy: the bronchial mucosa of the basal segment of the lower lobe of the right lung was slightly congested, the mucosa was smooth, there were many white viscous secretions in the lumen, and the interbronchial ridge was sharp, and no neoplasm or active bleeding was seen. The rest of the bronchi were unremarkable

**Fig. 3:**
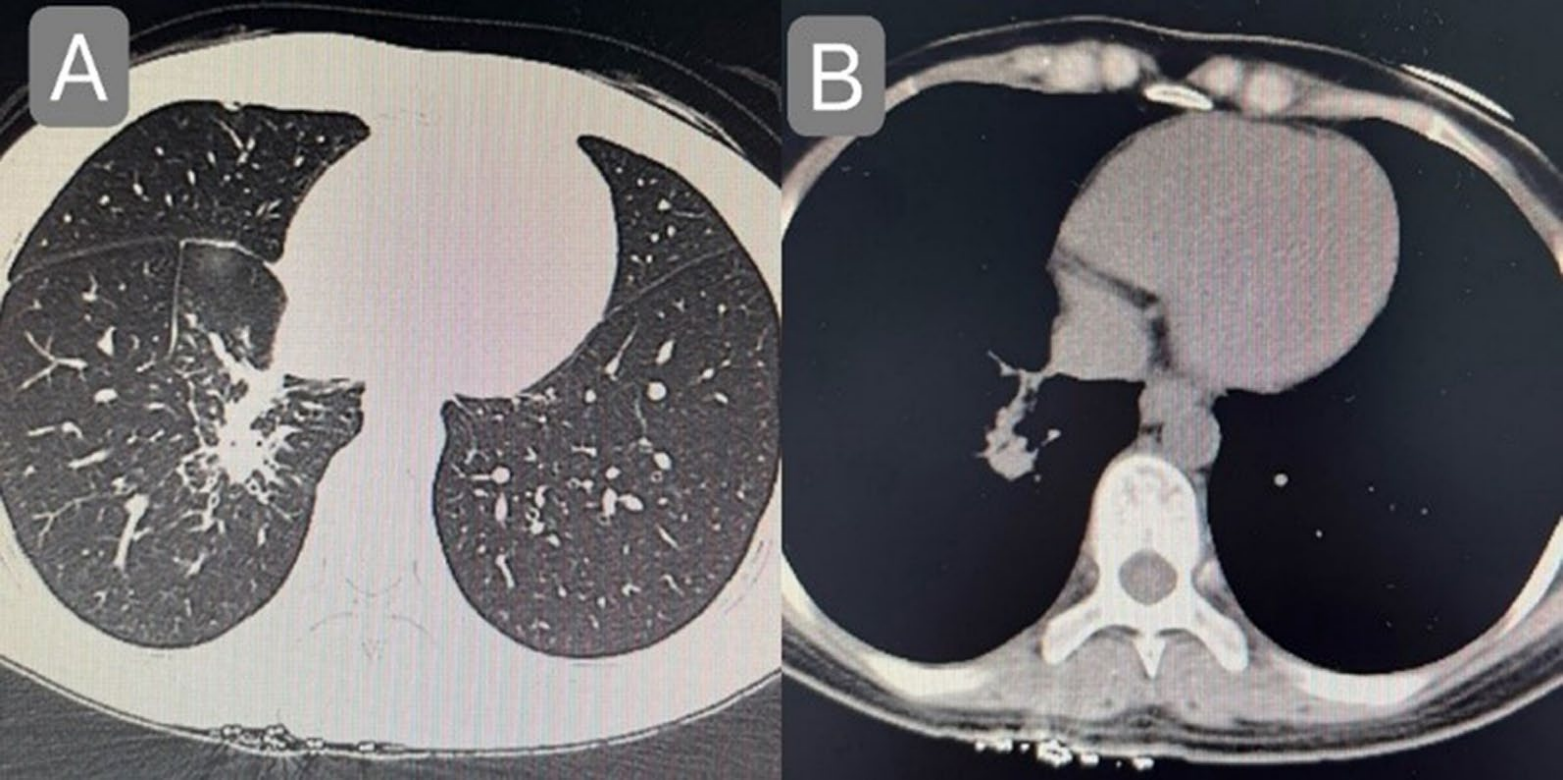
**A**, **B** There is a slight consolidation shadow in the right lower lobe near the hilum, compared with the previous imaging, the scope of the lesion is obviously reduced, and infectious disease is considered

The patient, a 29-year-old female, complained of cough and sputum that had aggravated for 1 month with fever for 5 days. The patient was engaged in civilian work in a township government building, and had no history of smoking, dust and other harmful environmental exposure. After anti-infective and symptomatic treatment at the local county hospital, the patient’s temperature decreased, but the cough and sputum did not resolve. She had a previous cesarean section and no history of other specific diseases. Initial vital signs were a temperature of 36.4 °C, heart rate of 77 beats/min, blood pressure of 104/72 mmHg, oxygen saturation (SpO_2_) of 92%, and respiratory rate of 20 breaths/min. Physical examination revealed hypopnea and scattered wet rales in the right lower lung, while other physical examination results showed no abnormalities. Subsequently, chest computed tomography (CT) was performed, which revealed a mass shadow in the right lower lobe near the hilar region, with obstructive atelectasis and obstructive pneumonia (Fig. 1). Routine blood tests showed significantly elevated leukocyte (11.88 × 10^9^/L), neutrophil (9.13 × 10^9^/L), platelet (425 × 10^9^/L), and C-reactive protein (77.74 mg/L) levels and mildly decreased hemoglobin levels. Sputum smear revealed gram-positive cocci (2+) and gram-negative bacilli (3+). Pulmonary function tests suggested mild restrictive ventilatory dysfunction, mild obstruction of small airway airflow, and mildly reduced ventilatory reserve function. To further clarify the diagnosis, enhanced CT was performed again after admission, and the results suggested a mass shadow in the lower lobe of the right lung near the hilar region (Fig. 1), suggesting a possible central lung cancer with obstructive pneumonia changes, with mediastinal and right hilar lymph node enlargement. Bronchoscopy revealed mild congestion of the bronchial mucosa in the basal segment of the lower lobe of the right lung, smooth mucosa, white mucous secretions in the lumen, sharp interstitial ridges, and no neoplastic growth or active bleeding (Fig. 2). The metagenomic next-generation sequencing (mNGS) test of alveolar lavage fluid showed *R. mannitolilytica*, sequence number 3271. Moreover, no cancer cells or *Mycobacterium tuberculosis* were detected. After a comprehensive evaluation, spherical pneumonia due to *Ralstonia solani* infection was considered. We used symptomatic treatments such as moxifloxacin for anti-infection, amiloride to dissolve sputum, compound ipratropium bromide to dilate the bronchi, and Suhuang Zhike Capsule (a widely used Chinese patent medicine with cough relieving and phlegm resolving properties) for evidence-based treatment. The patient’s clinical symptoms were significantly relieved after treatment, and repeat chest CT performed 10 days after admission showed significant absorption of the lesion (Fig. 3). The patient was subsequently discharged from the hospital and has shown no specific discomfort during follow-up to date.

## Discussion

Spherical pneumonia is clinically rare and more common in children than in adults, mainly because the pores of Kohn and the canals of Lambert in the lungs, which allow intra-alveolar communication, are poorly developed. Closely spaced connective tissue and smaller alveoli are more prone to dysplasia, and make it easy for lesions to accumulate once pneumonia develops. In addition, some pathogens have a strong affinity for alveolar epithelial cells, such as *Streptococcus pneumoniae*, and when infection occurs, alveolitis predominates, while bronchiolitis remains mild. In adults, it may also usually be due to defects in the development of intra-alveolar communication, or during the delayed resolution of lobar pneumonia, which may be termed focal organizing pneumonia [5]. In addition, imaging findings showed that spherical pneumonia was also closely related to the stage of the disease, and spherical pneumonia was easy to appear in following cases, such as early centrifugal diffusion of pneumonia; during the centripetal absorption of pneumonia through the alveoli; the development of pneumonia is limited after antibiotic treatment [4].

There are two characteristics that can help identify spherical pneumonia: first, a specific morphology that tends to appear on imaging; second, lung infection [6]. The imaging of spherical pneumonia has certain characteristics [7]. Firstly, spherical pneumonia mostly occurs in the back and near the pleura, with the diameter of the lesions generally being 1–7 cm, with an average of 4 cm; the density can be uneven, with higher density in the central area and lower density at the edge. Secondly, the edges of spherical lesions in adult patients and children over 8 years of age could show blurry shadows, while showing clear edges in children under 8 years of age. Thirdly, spherical pneumonia may have a pleural reaction, but pleural effusion is rare, and lymph node enlargement of hilum and mediastinum is rare. Finally, the chest CT signs of spherical pneumonia can be presented as a halo sign or a knife-cut sign. In addition, the lesions can show enhancement on enhanced CT, while the lesions can also show hypermetabolism and standard uptake value increase on PET-CT [8]. Although the halo sign is also one of the characteristics, it lacks specificity and can be seen in many diseases, such as invasive pulmonary aspergillosis, Wegener granuloma, pulmonary infarction, staphylococcus aureus pneumonia, etc. Although the knife cut sign is specific, the diagnosis of spherical pneumonia cannot only rely on imaging, but still needs evidence from etiology, clinical response, and even pathology. Therefore, the differential diagnosis should be the core for the diagnosis of spherical pneumonia. Spherical pneumonia is a pathological process in which the lung structures are not damaged or necrotic. Clinical diagnosis is difficult and often requires the differential diagnosis of lung cancer, tuberculosis spheres, inflammatory myofibroblastoma, malignant tumors, and granulomatous vasculitis.

In the present case, the patient’s chief complaint combined with the physical examination of the right lower lung indicating diminished breath sounds with wet rales and routine blood results led to the consideration of an infection in the lung. However, the possibility of a tumor or other types of diseases mentioned above could not be excluded because of the CT findings which revealed unexplained mass-like shadow changes in the lung. For further diagnosis, we performed enhanced CT and fibronectomy. Electronic bronchoscopy was used to clarify the inflammatory changes in the right lower bronchus, and no neoplasm was observed; thus, pulmonary malformation tumor and granulomatous polyangiitis were excluded. Fibronectomy and metagenomic next-generation sequencing (mNGS) test of lavage fluid showed *R. mannitolilytica,* and no cancer cells or *M. tuberculosis* were detected; thus, the possibility of the lesion being a lung cancer with tuberculosis sphere was excluded. Combined with the mass-like shadow in the chest lesion area and the differential diagnosis of each type, we ascertained the patient to have spherical pneumonia and administered anti-infective treatment. For treatment, we chose quinolones based on the patient’s sputum smear suggestive of gram-negative bacteria (3+) and the rare *R. mannitolilytica* in the alveolar lavage fluid. Quinolones are antimicrobially strong with a wide range of antibacterial activities, and are less likely to develop resistance. Among the commonly used quinolones, moxifloxacin has a high antibacterial success rate, and its broad antimicrobial spectrum makes it suitable for the treatment of intracellular gram-negative pathogens and atypical pathogens [9]. Therefore, moxifloxacin was chosen for one course of symptomatic supportive therapy. After 10 days, the patient’s symptoms significantly reduced, and CT reexamination showed significant absorption of the lesion. This allowed us to exclude inflammatory pseudotumors of the lung, that shows non-absorption of the lesion after anti-infective treatment, and clearly diagnose spherical pneumonia. This case suggests that during the diagnosis of spherical pneumonia, attention should be paid to the combination of symptoms and signs, and various examination tests should be performed to differentiate it from similar diseases before confirming the diagnosis.

To further summarize the clinical characteristics of spherical pneumonia, we searched PubMed, Web of Science, and Cochrane Library using the keywords “round pneumonia” and “spherical pneumonia”, and only 25 literature were reported in the past 20 years after screening [1–3, 5, 6, 8, 10–27], with a total of 29 cases reported (Table 1). The age of onset ranged from 4 months to 77 years, with 8 cases (27.5%) in minors under the age of 18 years, 8 (27.5%) in young adults aged 20–45 years, and 13 (45%) in middle-aged and older adults aged ≥ 45 years. The most common clinical symptoms of the disease were fever (79%), cough (62%), dyspnea (41%), and chest pain (28%), as well as sputum in 20% of the patients, including one with blood in the sputum. Other symptoms included gastrointestinal symptoms (14%), headaches (14%), myalgia (14%), and fatigue and weakness (14%). Other symptoms also included hemoptysis, hypoxemia, and arthralgia. The lesions varied in size, ranging from 20 to 80 mm. They were mainly located in the right lung, accounting for 66% of the total cases, including 12 in the right upper lobe (41%), 1 in the right middle lobe (3%), and 6 in the right lower lobe (21%). There were 5 cases of lesions in the left upper lung (17%), 5 in the left lower lung (17%), and 1 in the lingual segment (3%). Fourteen of the 29 cases had clearly detectable pathogens, including *S. pneumoniae*, *Enterobacter hormaechei, Rickettsia typhi,* and *Chlamydia pneumoniae.* The anti-infective treatment was effective in 28 cases, and no treatment measures were mentioned in one case. Broad-spectrum antibiotics were mostly used for anti-infective treatment, including quinolones, tetracyclines, penicillin, and other antibiotics. The lesions in the 29 patients were gradually absorbed and shrunk to disappear within 2–6 weeks, and the prognosis was good.

**Table 1:** Case reports of spherical pneumonia

	References	Author and year of publication	Age of the patient	Sex	Clinical presentation	Pathogens	Size and location	Treatment	Outcome
1	[1]	Köhne et al. (2012)	55 yo	Male	Fever, cough, tachypneic	Not available	A homogeneous mass in the left mid-lung zone is observed (left upper)	A 14-day course of ceftriaxone	The consolidation resolved on follow-up CT scan after two weeks
2	[2]	Gupta et al. (2019)	29 yo	Female	Fever, cough, breathlessness, right pleuritic chest pain	Not available	A 5.6 × 4.9 × 5.6 cm round mass-like opacity in the right upper lobe	Broad-spectrum antibiotics 14 days	Resolution of the lung mass
3	[3]	Camargo et al. (2008)	57 yo	Female	No complaints	Not available	A chest radiograph displayed a right lower-lobe mass	Not available	The process had resolved
4	[5]	Madhavan et al. (2014)	16 yo	Male	Fever, cough, tachypnoeic	Not available	A rounded opacity in the right upper zone with well-defined margin	Cefotaxime and azithromycin	Good clearance of the lesion
5	[6]	Liu et al. (2014)	7 yo	Male	Fever, dry cough, abdominalgia, decreased appetite, vomit, diarrhea	Streptococcus pneumoniae	A round-shaped opacity with clear margins in the left lower lobe and the retrocardiac region, 5.9 × 5.6 × 4.3 cm in size	Amoxicillin/clavulanate and azithromycin for 4 days,then cefibute and azithromycinv for 10 days	Complete resolution of the left lower lobe lesion
6	[8]	Shie et al. (2007)	75 yo	Male	Fever and intermittent hemoptysis	Streptococcus viridans	A 3-cm-diameter mass with irregular margins in the lingula abutting the pleura	A course of intravenous antibiotics	Resolution of the chest finding
7	[10]	Su et al. (2015)	25 yo	Male	Right anterior chest wall pain	Streptococcus pneumoniae	A 25 mm opacification over right upper lobe	Amoxicillin-clavulanate for 14 days	Regression of air-space opacification over right upper lobe
8	[11]	Harvey et al. (2014)	70 yo	Female	Fever, breathlessness, productive cough, tachypnoeic, hypoxaemic	Not available	A 60 mm mass in the right upper lobe	A course of co-amoxiclav and clarithromycin	Symptom and the right upper lobe abnormality resolution
9	[12]	AlOmran et al. (2021)	10 yo	Female	Fever, dry cough, decreased appetite	Streptococcus pneumoniae	2 well demarcated homogeneous lesions in the right upper and lower lobes	Penicillin and gentamicin for 3 days, then amoxicillin for 7 days	Resolving round opacities with complete resolution
10	[13]	Jiménez-Castillo et al. (2021)	64 yo	Male	Cough, exertional dyspnea	E. hormaechei	A round opacity in the right upper lobe	Ceftriaxone,clarithromycin, levofloxacin, imipenem/cilastatin	Died of septic shock
11	[14]	Yoshimura et al. (2015)	43 yo	Male	Fever, fatigue, headache	Rickettsia typhi	A nodular lesion and pleural effusion in the right lower lobe	Minocycline	Symptoms improved, lesions in the lung were diminished
12	[15]	Koinuma et al. (2019)	6 yo	Male	Fever, cough	C. pneumoniae	2 round opacities in the right lower lung field	Clarithromycin for 10 days	The gradual resolution of round pneumonia
13	[16]	Mahmood et al. (2014)	74 yo	Female	Dry cough, breathlessness	Pneumococcal	Rround consolidation with air bronchogram in the right lower lobe	Antibiotic therapy	Near-complete resolution of round consolidation
14	[17]	An et al. (2018)	77 yo	Male	Bloody sputum, fever	K. pneumoniae	An irregular opacity with lobulated borders in the right upper lobe	Antibiotic therapy	Right upper lung lesion narrowed and absorbed
15	[18]	Cunha et al. (2013)	50 yo	Male	Cough, fever, malaise, myalgias, breathlessness	Not available	A round opacity in the right upper lobe	Doxycycline for 6 weeks	Near complete resolution of round pneumonia
16	[19]	Çimen et al. (2015)	46 yo	Male	Cough, sputum, pain in left lower chest	Streptococcus pneumoniae	A 3 cm round homogeneous opacity in the left lower lung field	Moxifloxacin for 2 weeks	Biochemical parameters regressed and ABG came within the normal range. CXR showed complete resolution of the lingular RP
17	[19]	Çimen et al. (2015)	20 yo	Male	Cough, sputum and right flank pain	Not available	A 32 × 13 mm pleural-based consolidation, in the laterobasal and posterobasal segments of the right lower lobes, with air bronchograms	Levofloxacin	CT scan of thorax revealed regression of consolidation
18	[20]	Celebi et al. (2008)	4 mo	Male	Cough, tachypneic, tachycardic and febrile(fever)	Not available	A round homogenous lesion in the right upper lobe	Cefuroxime plus clindamycin for 1 week	The round lesion and infiltrate cleared radiographically within seven days
19	[20]	Celebi et al. (2008)	6 yo	Male	Fever and left upper abdominal pain	Not available	A 3 cm round lesion in the left upper lobe	A 14-day course of cefuroxime	A rapid resolution of clinical and radiographic findings
20	[20]	Celebi et al. (2008)	2 yo	Female	Tachypneic, tachycardic and febrile	Not available	A 3 cm round homogenous lesion in the right upper lobe	Cefuroxime plus clindamycin	Symptoms improved rapidly
21	[20]	Celebi et al. (2008)	7 yo	male	Fever and vomiting	Not available	A homogenous, smoothly marginated, 3 cm round lesion in the right upper lobe	Ampicillin-sulbactam	Clinical condition was improved The lesion had cleared almost totally
22	[21]	Jardim et al. (2003)	57 yo	Male	Cough, yellow phlegm, fever	Not available	A round homogenous lesion in the left upper lobe	Clarithromycin	Resolution of the lung mass
23	[22]	Núñez Viejo et al. (2010)	56 yo	Female	Cough, fever, chest pain	Streptococcus pneumoniae	Multiple pulmonary nodules	Levofloxacin	Symptoms improved and regression of nodules
24	[23]	Jiménez-Castillo et al. (2019)	40 yo	Male	Fatigue, fever, intermittent episodes of mild headache, dyspnea, dry cough	Pulmonary pneumocystis infection	A focal rounded opacification at the lower lobe of the left lung	Trimethoprim and sulfamethoxazole	The round lesion was not present after 4 days
25	[24]	Velasco-Tirado et al. (2012)	58 yo	Male	Fever, chills, oppressive headache and diffuse abdominal pain	R. typhi	A nodule of 2 cm in upper right lobe with adjacent pneumonitis	Doxycycline	Symptoms improved, lesions in the lung were diminished
26	[24]	Velasco-Tirado et al. (2012)	20 yo	Male	Fever, dry cough, arthralgias, myalgias, headache, sweating and vomiting,macular rash	R. typhi	A nodular lesion in middle lobe	Doxycycline	A CR obtained 14 days after diagnosis was normal
27	[25]	Kara et al. (2010)	26 yo	Female	Fever and myalgia	Not available	A spherical pattern with a homogeneous 4.5 × 4.5 cm diameter opacification on the right middle lobe	Clarithromycin for 10 days	Symptom-free
28	[26]	Violante-Cumpa et al. (2019)	44 yo	Female	Asthenia, adynamia, dyspnea and orthopnea	Not available	A round opacity with an air bronchogram in the superior lobe of the left lung with measurements of 8.6 × 5.6 cm	Ceftriaxone and clarithromycin for 7 days	Clinical improvement
29	[27]	Durning et al. (2003)	58 yo	Female	Cough, fever, dyspnea, and vague left upper abdominal pain	Not available	A 4 cm round mass in the left lower lobe	A 14-day course of levofloxacin	A rapid resolution of clinical and radiographic findings

yo, years old; mo, month old

The common pathogens of spherical pneumonia are similar to those of community-acquired pneumonia, with *S. pneumoniae* being considered common. However, in recent years, the detection rate of atypical pathogens has increased. Mycoplasma and *Coxiella burnetii* are known to be common pathogens, and some studies have suggested that Q fever is the main cause of spherical pneumonia [4]. In addition, *Enterobacter cholerae*, *Rickettsia typhi*, and SARS-Co-V2 can also cause spherical pneumonia [28]. However, spherical pneumonia due to *R. mannitolilytica* infection has not yet been reported. *R. mannitolilytica* is a gram-negative bacterium (family *Burkholderiaceae*, genus *Ralstonia*), which mainly survives in water sources but also in low nutrient environments. It was first discovered by Yabuuchi in Japan in 1995 [29]. There are currently three clinically relevant species, including *R. mannitolilytica*, *Ralstonia pickettii*, and *Ralstonia insidiosa* [30]. In 2001, *R. mannitolilytica* was classified as a new species of the genus *Ralstonia,* based on 16S rRNA gene sequence analysis [31]. *R. mannitolilytica* was first isolated in China from a patient with chronic obstructive pulmonary disease [32]. In 2015, the whole-genome sequence analysis of *R. mannitolilytica* strain MRY14-0246, which carries intrinsic OXA-443/OXA-22-like and OXA-444/OXA-60-like β-lactamase genes and is resistant to meropenem, was performed [33]. *R. mannitolilytica* is a rare opportunistic causative agent of intra-hospital infections. *R. mannitolilytica* infects humans predominantly through water sources and affects mainly immunocompromised patients. There are few national and international reports, and there are current case reports of cystic fibrosis with infection, elderly hospital-acquired pneumonia, bronchiectasis, chronic obstructive pulmonary disease, bacteremia, solid cancer, abdominal dialysis tube infection, neonatal, hematologic, renal transplantation, diabetes mellitus, scleroderma, meningitis, peritonitis, osteomyelitis, abdominal blood collection infection, and urinary tract infection. Elderly patients, those with multiple underlying diseases, those using broad-spectrum antibacterial drugs, and those receiving invasive mechanical ventilation are at an increased risk of infection with this organism [32, 34–45]. Although previous reports showed that the infection caused by *R. mannitolilytica* is more common in immunocompromised groups, the report of *R. mannitolilytica* infection in the normal immunity population is rare, and the specific mechanism is still unclear. It has been shown that *R. mannitolilytica* has the hemH gene encoding protoporphyrin ferrous chelatase, and protoporphyrin ferrochelatase may act as an important virulence factor of this bacteria [46]. The downregulation of protoporphyrin ferrous chelatase expression leads to the accumulation of protoporphyrin substrates and the production of large amounts of reactive oxygen species such as singlet oxygen and peroxide radicals through phototoxic reactions, leading to tissue cell damage and triggering inflammation [47]. Many of the cases of infection with *R. mannitolilytica* are due to contaminated solutions, including water for injection, respiratory solutions, saline solutions, and sterile drug solutions [48]. The outbreak of *R. mannitolilytica* infections in the hospital is typically associated with contaminated medical supplies or instruments. An investigation revealed colonization with *R. mannitolilytica* of two in 15 patients and contamination of components of five of six humidifying respiratory therapy device [49]. However, in most cases, the source is unknown. Although the young patient reported in this article had no basic diseases and had normal immunity, the cause of infection with *R. mannitolilytica* is still unclear. The spherical lesion on chest CT could stem from the fact that the patient has received anti-infection treatment outside the hospital. Previous studies have shown that pneumonia caused by *R. mannitolilytica* can be manifested on chest CT as patchy exudative shadows, consolidation, pleural effusion, pulmonary cavity or bronchiectasis [50, 51]. Currently, the pathological manifestations of pneumonia caused by *R. mannitolilytica* have not been reported. Although automatic bacteria identification instrument (the Mérieux VITEK MS mass spectrometer) and 16S rRNA detection are the main identification tools, it is difficult to detect *R. mannitolytica* in routine sputum culture. Therefore, clinical identification of *R. mannitolilytica* is challenging.

mNGS, which does not rely on traditional microbial culture and directly sequences nucleic acids in clinical samples at high throughput, can rapidly and objectively detect a wide range of pathogenic microorganisms (including viruses, bacteria, fungi, and parasites) in clinical samples. mNGS is a more sensitive technique than traditional pathogen culture, with evidence from studies showing that mNGS has a diagnostic sensitivity of 88.89% for all pathogens and 83.37% for negative predictive value [52]. The diagnostic process of the case reported in this study showed that *R. mannitolilytica* causing spherical pneumonia could be clinically identified using mNGS testing.

In the treatment of spherical pneumonia, antibiotic selection should be based on the culture results. When immunocompromised patients are infected by *R. mannitolilytica*, the disease can progress rapidly into sepsis or even multiple organ dysfunction syndrome; therefore, the sensitive antibiotics should be administered as early as possible. According to the literature, *R. mannitolilytica* is often resistant to multiple antibiotics [53]. There are no treatment guidelines or drug susceptibility recommendation for this pathogen. Previous studies showed that *R. mannitolilytica* was resistant to aztreonam, cefoperazone and meropenem, but sensitive to cotrimoxazole, quinolones, third and fourth generation cephalosporins, and cefoperazone/sulbactam [54, 55]. Owing to variable resistance to multiple antibacterial drugs and the lack of interpretative criteria for drug susceptibility in *R. mannitolilytica*, physicians need to use drugs based on clinical experience. In the future, the research on *R. mannitolilytica* needs to be further strengthened.

## Conclusion

Spherical pneumonia caused by *R. mannitolilytica* has not been previously reported. For clinical diagnosis, we could combine signs and symptoms and differentiate it from other similar diseases. The mNGS method can be used for strain identification when clinical diagnosis is difficult. *R. mannitolilytica* is highly resistant to various antimicrobial drugs, and clinicians need to use drugs according to clinical experience, and make timely efficacy assessment and adjustment of drugs.

## Data Availability

Not applicable.

## Abbreviations

*R. mannitolilytica*: *Ralstonia mannitolilytica*
mNGS: Metagenomic next-generation sequencing
CT: Chest computed tomography
